# Patient-reported impact of symptoms in adrenoleukodystrophy (PRISM-ALD)

**DOI:** 10.1186/s13023-024-03129-6

**Published:** 2024-03-19

**Authors:** Anika Varma, Jennifer Weinstein, Jamison Seabury, Spencer Rosero, Nuran Dilek, John Heatwole, Charlotte Engebrecht, Shaweta Khosa, Kaitlin Chung, Asif Paker, Amy Woo, Gregory Brooks, Chan Beals, Rohan Gandhi, Chad Heatwole

**Affiliations:** 1 Center for Health + Technology, University of Rochester, 265 Crittenden Blvd, CU 420694, Rochester, NY 14642 USA; 2 Department of Neurology, University of Rochester, 601 Elmwood Ave, Box 673, Rochester, NY 14642 USA; 3 Cornell University, Ithaca, NY 14850 USA; 4 SwanBio Therapeutics, 150 Monument Rd, Bala Cynwyd, PA 19004 USA; 5 Autobahn Therapeutics, 9880 Campus Point Drive, San Diego, CA 92121 USA

**Keywords:** Adrenoleukodystrophy, Adrenomyeloneuropathy, Symptom, Disease burden, Qualitative research, Patient interview, Cross-sectional study, Patient-reported

## Abstract

**Background:**

Adrenoleukodystrophy (ALD) is a multifaceted, X-linked, neurodegenerative disorder that comprises several clinical phenotypes. ALD affects patients through a variety of physical, emotional, social, and other disease-specific factors that collectively contribute to disease burden. To facilitate clinical care and research, it is important to identify which symptoms are most common and relevant to individuals with any subtype of ALD.

**Methods:**

We conducted semi-structured qualitative interviews and an international cross-sectional study to determine the most prevalent and important symptoms of ALD. Our study included adult participants with a diagnosis of ALD who were recruited from national and international patient registries. Responses were categorized by age, sex, disease phenotype, functional status, and other demographic and clinical features.

**Results:**

Seventeen individuals with ALD participated in qualitative interviews, providing 1709 direct quotes regarding their symptomatic burden. One hundred and nine individuals participated in the cross-sectional survey study, which inquired about 182 unique symptoms representing 24 distinct symptomatic themes. The symptomatic themes with the highest prevalence in the overall ALD sample cohort were problems with balance (90.9%), limitations with mobility or walking (87.3%), fatigue (86.4%), and leg weakness (86.4%). The symptomatic themes with the highest impact scores (on a 0–4 scale with 4 being the most severe) were trouble getting around (2.35), leg weakness (2.25), and problems with balance (2.21). A higher prevalence of symptomatic themes was associated with functional disability, employment disruption, and speech impairment.

**Conclusions:**

There are many patient-relevant symptoms and themes that contribute to disease burden in individuals with ALD. These symptoms, identified by those having ALD, present key targets for further research and therapeutic development.

**Supplementary Information:**

The online version contains supplementary material available at 10.1186/s13023-024-03129-6.

## Background

Adrenoleukodystrophy (ALD) is an X-linked genetic condition caused by mutations in an ATP-binding cassette gene (ABCD1) that encodes an ABC transporter, which is involved in transporting very long chain fatty acids (VLCFs) to the peroxisome for degradation [1–6]. As a result of the mutations (more than 600 known pathogenic variants), VLCFs are not able to be properly processed, and they accumulate in the tissues, causing a host of issues that present with varying phenotypes according to age, sex, and clinical characteristics [7, 8]. The three core clinical phenotypes of ALD are (1) a slowly progressive myeloneuropathy (adrenomyeloneuropathy or AMN); (2) a rapidly progressive leukodystrophy (cerebral ALD); and (3) primary adrenal insufficiency (Addison’s disease) [9, 10]. Both women and men can be affected by AMN (also called symptomatic ALD in women) [10]. Cerebral ALD and Addison’s disease predominantly occur in men (women affected at < 1%) [10]. Additionally, women with ALD may remain completely asymptomatic throughout their lives despite being gene carriers (termed asymptomatic women with ALD) [10]. Although ALD can be detected through newborn screening (genetic testing and/or biochemical testing), age of symptom onset is variable, ranging from childhood-adulthood for males presenting with cerebral ALD and/or adrenal insufficiency to adulthood for males and females presenting with AMN [10, 11].

As a whole, ALD is recognized as the most common peroxisomal disorder, affecting approximately 1 in 16,800 in the U.S. (includes children and adults; symptomatic and asymptomatic men and women) [6].

Clinical manifestations of ALD depend on the specific phenotype associated with the condition. The most common symptoms of AMN include weakness and spasticity in the legs, abnormal sphincter control, neurogenic bladder, sexual dysfunction, numbness, and pain [12]. Cerebral ALD may show up as learning disabilities, behavioral abnormalities, cognitive decline, impaired vision and/or auditory discrimination, and seizures [13, 14]. Addison’s disease may cause symptoms such as fatigue, muscle weakness, low mood/mild depression, nonspecific gastrointestinal issues, vomiting, weakness, and headaches [15–17].

While a few treatment options exist for individuals with ALD, again depending on the particular phenotype (spasticity-reducing medications and neuropathic pain medications for AMN, hematopoietic stem cell transplant for cerebral ALD, and glucocorticoid replacement for Addison’s disease), there are no cures [9, 18]. In order to facilitate clinical care and identify potential targets for therapeutic intervention, it is important to better understand the most salient symptoms to patients from the perspective of those with ALD (including all phenotypes).

In this study, we collected data from semi-structured interviews with individuals with ALD and subsequently conducted a large cross-sectional study to identify the most prevalent and impactful symptoms to individuals with this disease, including all subtypes. This information will help guide researchers, clinicians, and therapy developers to better care for and treat all patients with ALD.

## Methods

### Study participants

Participants for this study were recruited from the following organizations: ALD Connect (active in the U.S. and Canada); the United Leukodystrophy Foundation (active in the U.S.); Alex—The Leukodystrophy Charity (active in the U.K.); Fundación Lautaro te Necesita—Leukodystrophy Foundation (active in South America); The Leukodystrophy Resource Research Organization (LRRO) (active in Australia); Royal Children’s Hospital and Massimo’s Mission (active in Australia); and Leukodystrophy Australia (active in Australia). Eligible participants were those who: (1) were age 18 or older; (2) had a general diagnosis of ALD or a specific diagnosis to include AMN, cerebral ALD, Addison’s disease, and/or asymptomatic women with ALD; and, (3) were able to speak, read, and understand English.

All study activities were approved by the University of Rochester Institutional Review Board, and participants were required to provide informed consent prior to taking part in interviews and/or the cross-sectional study. Interviews were conducted between May 17, 2021 and July 23, 2021, and the subsequent cross-sectional study was conducted between November 2, 2021 and January 17, 2022.

### Study design

#### ALD qualitative interviews

We conducted 30–60 min semi-structured qualitative interviews with individuals with all types of ALD to identify the symptoms that have the greatest impact on their lives. Potential participants were informed of the purpose of the study, the risks and benefits, and their rights prior to providing consent via phone.

Three clinical research coordinators (SR, JW, JS) conducted the participant interviews. The interviewers asked open-ended questions regarding the physical, mental, emotional, social, and everyday health of the participants. For example, participants were asked “which symptoms have the greatest impact on a person’s quality-of-life or disease burden?,” “how is a person with ALD/AMN affected physically and emotionally by the disease,” and “what are the little things that are affected by and important to people with ALD/AMN?,” among other questions. The interviews were recorded via Zoom (a HIPAA-compliant conferencing software), transcribed, and coded by the research team (SR, JW, JS, AV, and CH) to analyze direct participant quotes, pinpoint unique symptoms, and classify symptoms into symptomatic themes (groups of related symptoms). This coding process followed a qualitative framework technique and multi-investigator consensus approach that has been used in previous studies of other diseases [19–27]. We conducted interviews until data saturation was reached [28].

#### International cross-sectional study of individuals with ALD

We constructed a survey that included the symptoms and symptomatic themes that were brought up by participants repeatedly during qualitative interviews as well as those that have been identified by experts in this field as being important to patients with any phenotype of ALD. We implemented this survey in an international cross-sectional study with individuals with all types of ALD to determine the prevalence and relative importance of these symptoms and themes. The survey was administered via REDCap (a HIPAA-compliant electronic data capture system) and was accessible to participants through a public survey link distributed by partnering recruitment organizations. Participants were first directed to read a patient information letter and General Data Protection Legislation (GDPR) notice (for participants from the European Union or United Kingdom). Participants then completed an online consent form and answered demographic and clinical questions prior to taking the symptom survey. The symptom survey inquired about 182 symptoms representing 24 symptomatic themes. For each symptom question, individuals were asked, “how much does the following impact your life now?” They were presented with a 6-point Likert-type scale to record their responses; the scale consisted of the following options: (1) I don’t experience this; (2) I experience this but it does not affect my life; (3) It affects my life a little; (4) It affects my life moderately; (5) It affects my life very much; (6) It affects my life severely. Individuals had the option to decline to answer any question. At the end of the survey, participants were asked to list and rank the impact of any other symptoms that were not included on the survey.

### Statistical analysis

Participants who met the inclusion criteria and who completed at least 1 demographic question and 1 symptom question on the cross-sectional survey were included in the data analysis. We used the data from the cross-sectional study to calculate the prevalence and impact of each symptom and symptomatic theme. Prevalence was calculated as the number of participants who experienced a symptom (options 2–6 on the Likert scale) normalized by the total number of participants who responded to the symptom question. Impact scores, on a scale of 0–4, were computed by assigning numerical values to each of the rating options on the Likert scale for all participants who reported experiencing the symptom: 0 = I experience this but it does not affect my life; 1 = It affects my life a little; 2 = It affects my life moderately; 3 = It affects my life very much; 4 = It affects my life severely.

Population impact (PIP) scores, on a scale of 0–4, were calculated by multiplying the prevalence, of the symptom by the average life impact score of the symptom. A score of 0 corresponded to no impact on the population, whereas a score of 4 corresponded to the highest possible impact to the population. The methods performed here have been described and validated previously for other diseases [19–27].

In addition to ascertaining the prevalence and importance of symptoms and themes in our sample, we compared the prevalence of the symptomatic themes in predetermined subcategories based on age (above mean vs. equal to/below mean); sex (male vs. female); education level (grade school, high school, technical degree, or none vs. college, master’s, or doctorate); employment status (working full-time, working part-time, or stay-at-home parent vs. on disability or not working/not on disability; excluding students, retired individuals, others); disability status (on disability vs. all other employment categories not on disability); and has ALD impacted employment status (yes vs. no). Data was also categorized based on other, disease-specific criteria, specifically number of years since first noticed symptoms (above mean vs. equal to/below mean); diagnosis of AMN (yes vs. no); diagnosis of cerebral ALD (yes vs. no); diagnosis of Addison’s disease/adrenal insufficiency (yes vs. no); ambulatory status (walk independently vs. use a cane, crutches, walker, or motorized scooter); speech status (talk clearly vs. speech change); functional ability (no symptoms, no significant disability vs. slight disability, moderate disability, moderately severe disability, severe disability); and hours of home health aide per week (none vs. some aide). Fisher exact tests were used to compare the prevalence of each symptomatic theme between groups. These tests were exploratory and are reported for descriptive purposes only. To correct for multiple comparisons, the Benjamini–Hochberg procedure was used with a false discovery rate of 0.05 and 336 test statistics. As outlined by this method, the 336 *p* values were sorted from smallest to largest and the largest value of i such that *p*(i) ≤ 0.05 i/336 was determined. The null hypotheses associated with the *p* values *p*(1), …, *p*(i) were rejected, resulting in 56 “discoveries.”

## Results

### ALD qualitative interviews

We performed 17 interviews with individuals with ALD, including all phenotypes (AMN, cerebral ALD, Addison’s disease, and asymptomatic women with ALD). The interviewees consisted of 15 men (88.2%) and 2 women (11.8%) with ALD, and the ages ranged from 23 to 73 years with the mean age being 48 ± 13 years. The demographics of these participants are provided in Table 1. Through these interviews, we obtained 1709 direct quotes regarding patient-perceived symptoms of importance. From these quotes, 182 unique symptoms were extracted and grouped into 24 symptomatic themes. These themes were limitations with mobility or walking; problems with balance; inability to do activities; trouble getting around; leg weakness; pain; stiffness; fatigue; gastrointestinal issues; decreased satisfaction in social situations; emotional issues; communication difficulties; difficulty thinking; impaired sleep or daytime sleepiness; back, chest, or abdominal weakness; problems with shoulders or arms; numbness; choking or swallowing issues; abnormal movements; problems with hands or fingers; breathing difficulties; seizures; impaired vision; and difficulty hearing.

**Table 1:** Participant demographics for ALD interviews

Interviews completed, n	17
Sex, n (%)	
Male	15 (88.2)
Female	2 (11.8)
Race, n (%)	
White	16 (94.1)
Omitted	1 (5.9)
Hispanic or Latino, n (%)	
Yes	1 (5.9)
No	16 (94.1)
Age in years	
Mean ± 1 SD	48 ± 13
Range	23–72
Age at diagnosis in years	
Mean ± 1 SD	30 ± 17
Range	2 to 62
Ambulatory status, n (%)	
Fully ambulatory, no assistance	3 (17.6)
Ambulatory with canes/assistance	9 (53.0)
Non-ambulatory/wheelchair	5 (29.4)
U.S. states represented, n	8

Multiple-choice options that were not selected by any participant have been omitted for conciseness

Percents have been normalized for missing responses

### International cross-sectional study of individuals with ALD

A total of 158 participants responded to our cross-sectional survey with 109 respondents meeting our inclusion criteria for data analysis. This sample comprised 47 men (43.1%) and 62 women (56.9%), who represented a range of ages from 18 to 83 years with a mean age of 51 ± 17 years. The majority of participants identified as white (98 people; 89.9%) and non-Hispanic/Latino (94 people; 86.2%). Participants represented 16 countries, spanning the continents of North America, South America, Europe, Asia, and Australia. One Canadian province (Ontario) and 21 U.S. states were represented.

In our sample cohort, 59 people (54.1%) reported being diagnosed with general ALD; 71 people (65.1%) with AMN; 18 people (16.5%) with the cerebral form of ALD; and 35 people (32.1%) with Addison’s disease. These categories were not mutually exclusive. The average number of years since symptom onset was 15 ± 11 years, and the average number of years since diagnosis was 16 ± 11 years.

**Fig. 1:**
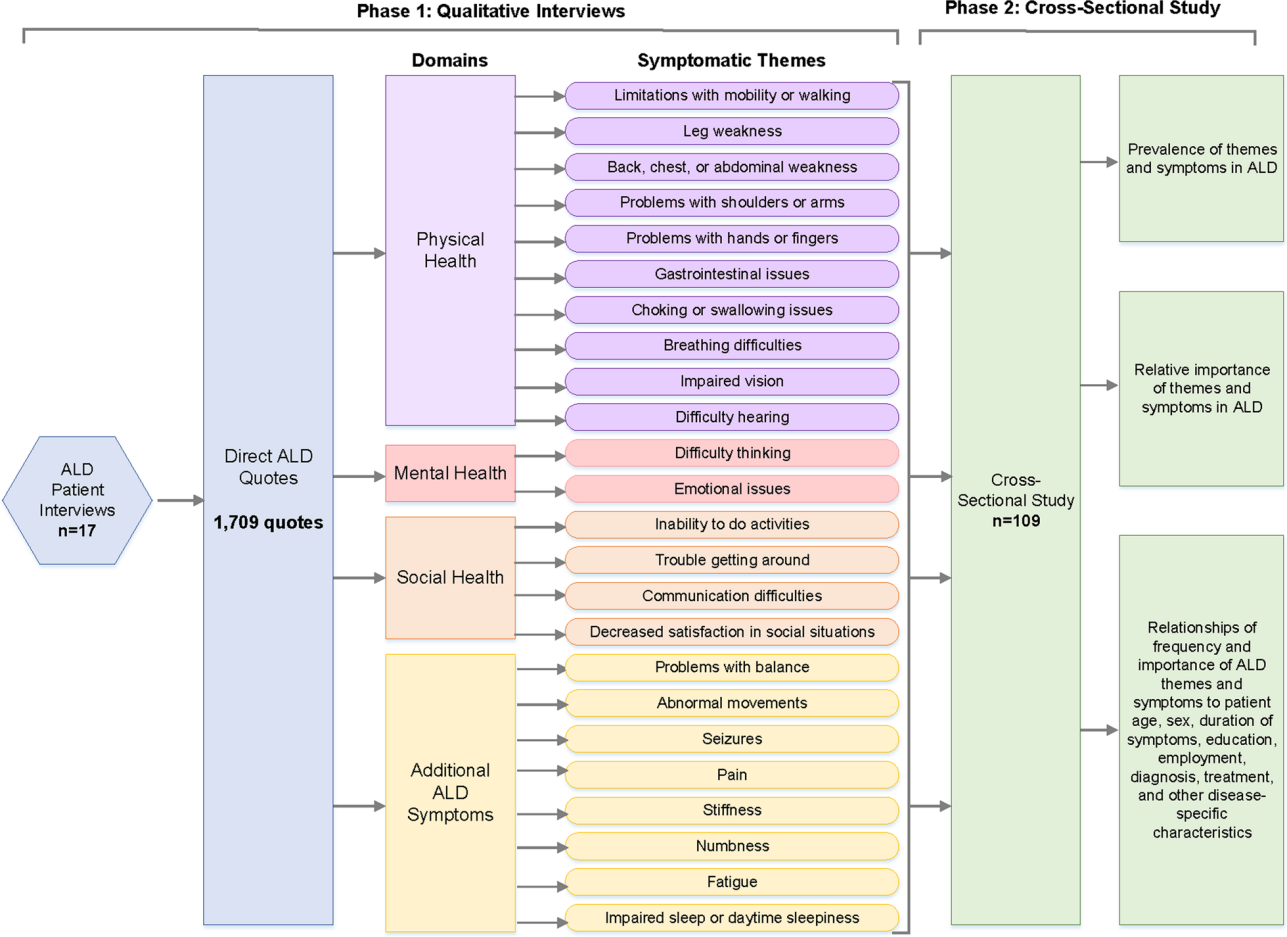
Overview of study activities to identify symptoms of importance to individuals with ALD

Table 2 provides additional details regarding the demographics of participants in the cross-sectional study. Figure 1 provides a complete outline of our study activities.

**Table 2:** Participant demographics for ALD cross-sectional study

Cross-sectional study participants, n	109
Sex, n (%)	
Male	47 (43.1)
Female	62 (56.9)
Age in years	
Mean ± 1 SD	51 ± 17
Range	18–83
Race, n (%)	
Asian	3 (2.8)
Black/African American	1 (0.9)
White	98 (89.9)
Other	7 (6.4)
Hispanic or Latino, n (%)	
Yes	15 (13.8)
No	94 (86.2)
Country, n (%) (16 total countries represented)	
United States	54 (49.5)
Canada	5 (4.6)
Australia	10 (9.7)
United Kingdom	16 (14.7)
France	2 (1.8)
Argentina	7 (6.4)
Bolivia	2 (1.8)
Chile	1 (0.9)
India	1 (0.9)
Iran	1 (0.9)
Ireland	3 (2.8)
Mexico	1 (0.9)
New Zealand	3 (2.8)
Poland	1 (0.9)
South Korea	1 (0.9)
Sweden	1 (0.9)
U.S. states represented, n	21
Canadian provinces represented (Ontario), n	1
Employment status, n (%)	
Employed full-time	29 (26.6)
Employed part-time	9 (8.3)
On disability	16 (14.7)
Not working/not on disability	8 (7.3)
Retired	30 (27.5)
Student	6 (5.5)
Stay-at-home parent	1 (0.9)
Self-employed	7 (6.4)
Other	3 (2.8)
Has ALD impacted your employment status or choice?, n (%)	
Yes	60 (55.1)
No	40 (36.7)
I don't know	8 (7.3)
Prefer not to answer	1 (0.9)
Highest level of education, n (%)	
Grade school	2 (1.8)
High school	29 (26.6)
Technical degree	19 (17.5)
College	33 (30.3)
Master's or Doctorate	24 (22.0)
None	2 (1.8)
Has ALD prevented you from pursuing additional education?, n (%)	
Yes	18 (16.5)
No	87 (79.8)
I don't know	4 (3.7)
Marital status, n (%)	
Married	61 (56.0)
Single	26 (23.9)
Widowed	3 (2.7)
Divorced	13 (12.0)
Separated	3 (2.7)
Registered partnership	3 (2.7)
Has ALD impacted your marital status or decision to pursue relationships?, n (%)	
Yes	35 (32.1)
No	70 (64.2)
I don't know	4 (3.7)
Diagnosed with ALD?, n (%)	
Yes	59 (54.1)
No	43 (39.5)
I don't know	7 (6.4)
Diagnosed with AMN?, n (%)	
Yes	71 (65.1)
No	27 (24.8)
I don't know	11 (10.1)
Diagnosed with cerebral form of ALD?, n (%)	
Yes	18 (16.5)
No	81 (74.3)
I don't know	10 (9.2)
Diagnosed with Addison's disease?, n (%)	
Yes	35 (32.1)
No	71 (65.1)
I don't know	3 (2.8)
Ever been in adrenal crisis?, n (%)	
Yes	22 (20.2)
No	82 (75.2)
I don't know	5 (4.6)
Years since diagnosis	
Mean ± 1 SD	16 ± 11
Range	1–40
Years since first noticed symptoms	
Mean ± 1 SD	15 ± 11
Range	0–50
Ever misdiagnosed?, n (%)	
Yes	38 (34.9)
No	68 (62.4)
I don't know	3 (2.7)
Ambulation, n (%)	
Walk independently without assistance	50 (45.9)
Primarily use a cane or crutches	34 (31.2)
Primarily use a walker	10 (9.2)
Use a wheelchair or motorized scooter sometimes and walk sometimes	7 (6.4)
Primarily use a wheelchair or motorized scooter	8 (7.3)
Hours of home health aide per week, n (%)	
None	75 (68.8)
1–5 h	15 (13.8)
6–10 h	6 (5.5)
16–20 h	3 (2.7)
Greater than 20 h	10 (9.2)
Speech, n (%)	
Talk clearly and have no changes in speech	89 (81.7)
Some speech changes	17 (15.6)
Impaired speech, and people occasionally ask to repeat words or phrases	2 (1.8)
Impaired speech that is often not understood by others	1 (0.9)
Positive genetic test for ABCD1 gene mutation?, n (%)	
Yes	89 (81.6)
No	5 (4.6)
No genetic testing	11 (10.1)
I don't know	4 (3.7)
Functional ability, n (%)	
No symptoms	4 (3.7)
No significant disability	26 (23.8)
Slight disability	32 (29.4)
Moderate disability	29 (26.6)
Moderately severe disability	18 (16.5)
Ever received bone marrow or stem cell transplant?, n (%)	
Yes	5 (4.6)
No	104 (95.4)
Current treatments, n (%)^a^	
Hormone replacement, steroid medications, or corticosteroids	34 (31.2)
High dose antioxidants (OTC)	4 (3.7)
Lorenzo's oil	2 (1.8)
Spasticity-reducing medications (Baclofen, Tazanidine, Botox, etc.)	33 (30.3)
Neuropathic pain medications or anti-epileptic medications (Neurontin e.g. Gabapentin)	32 (29.4)
Medications for overactive bladder or bowel	21 (19.3)
Cannabidiol (CBD)	15 (13.8)
Anti-depressants or anti-anxiety medications	24 (22.0)
Physical therapy	32 (29.4)
None	20 (18.4)

Multiple-choice options that were not selected by any participant have been omitted for conciseness

Percents have been normalized for missing responses

^a^Percents may not add up to 100% because some individuals receive multiple treatments

### Prevalence of symptomatic themes and symptoms

Of the 24 symptomatic themes assessed in the cross-sectional survey, the most prevalent ones in our sample cohort were problems with balance (90.8%), limitations with mobility or walking (87.2%), fatigue (86.2%), and leg weakness (86.2%). The most frequently occurring individual symptoms were fear of disease progression (91.8%), difficulty getting up from the floor or ground (90.1%), difficulty running (89.6%), and fatigue after physical activity (89.1%). Additional file 1: Table S1 provides the prevalence of all symptomatic themes and symptoms.

### Life impact of symptomatic themes and symptoms

The symptomatic themes with the highest average impact scores (on a scale of 0–4) from the cross-sectional survey were trouble getting around (2.34), leg weakness (2.24), problems with balance (2.21), inability to do activities (2.12), and limitations with mobility or walking (2.12). The most impactful individual symptoms were difficulty playing sports (3.06), difficulty running (2.94), difficulty riding a bike (2.89), and difficulty dancing (2.82). Additional file 1: Table S1 provides the average impact of all symptomatic themes and symptoms.

**Fig. 2:**
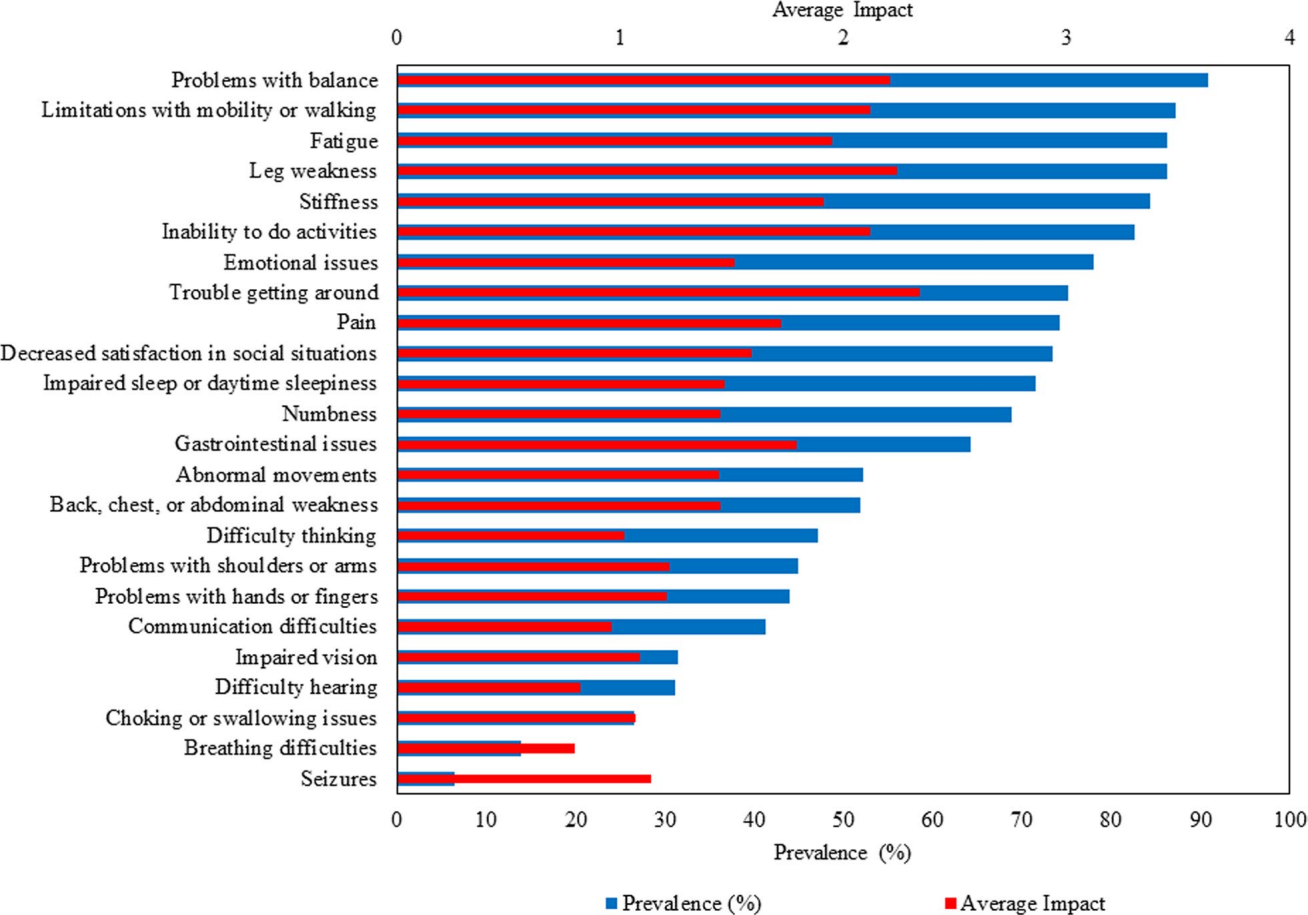
Prevalence and mean impact of symptomatic themes from ALD cross-sectional study

The prevalence and average impact of the 24 symptomatic themes that were asked about in the cross-sectional survey are shown in Fig. 2. Blue bars indicate prevalence (%), and red bars indicate average impact.

### Population impact (PIP) of symptomatic themes and symptoms

**Fig. 3:**
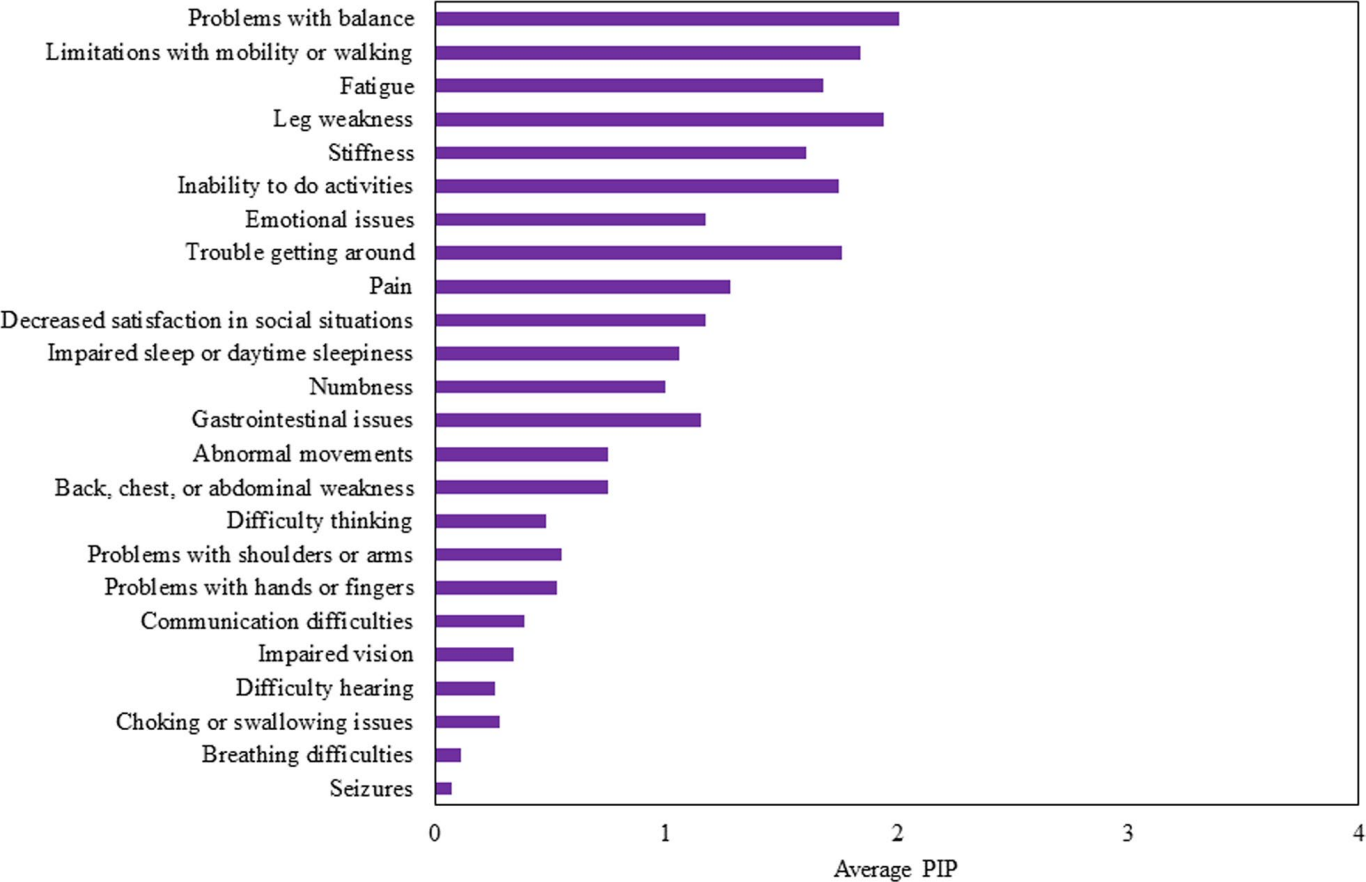
Population impact (PIP) of symptomatic themes from ALD cross-sectional study

The symptomatic themes with the largest PIP scores were problems with balance (2.01), leg weakness (1.94), limitations with mobility or walking (1.84), and trouble getting around (1.76). Difficulty running (2.63), difficulty playing sports (2.59), difficulty riding a bike (2.29), and fear of disease progression (2.21) were the individual symptoms with the highest PIP. The PIP values for the 24 symptomatic themes are shown in Fig. 3.

### Analysis of symptomatic themes by demographic category

The prevalence of several symptomatic themes differed by demographic and clinical subgroups, as displayed in Table 3.

**Table 3:** Prevalence of symptomatic themes in ALD for the overall sample (n = 109) and subgroups of individuals with ALD

	Overall prevalence (%) of full sample (n = 109)
*A: Theme*	
Limitations with mobility or walking	87.2
Problems with balance	90.8
Inability to do activities	82.6
Trouble getting around	75.2
Leg weakness	86.2
Pain	74.3
Stiffness	84.4
Fatigue	86.2
Gastrointestinal issues	64.2
Decreased satisfaction in social situations	73.4
Emotional issues	78.0
Communication difficulties	41.3
Difficulty thinking	47.2
Impaired sleep or daytime sleepiness	71.6
Back, chest, or abdominal weakness	51.9
Problems with shoulders or arms	45.0
Numbness	68.8
Choking or swallowing issues	26.6
Abnormal movements	52.3
Problems with hands or fingers	44.0
Breathing difficulties	13.9
Seizures	6.5
Impaired vision	31.5
Difficulty hearing	31.2

	Age (years)		
	Prevalence (%)		
	Above mean (> 51 years) (n = 61)	Equal to or below mean (≤ 51 years) (n = 48)	*p* value
*B: Theme*			
Limitations with mobility or walking	95.08	77.08	0.0080*
Problems with balance	96.72	83.33	0.0209*
Inability to do activities	86.89	77.08	0.2098
Trouble getting around	80.33	68.75	0.1855
Leg weakness	90.16	81.25	0.2627
Pain	81.97	64.58	0.0482*
Stiffness	90.16	77.08	0.0695
Fatigue	88.52	83.33	0.5769
Gastrointestinal issues	67.21	60.42	0.5471
Decreased satisfaction in social situations	70.49	77.08	0.5155
Emotional issues	73.77	83.33	0.2544
Communication difficulties	39.34	43.75	0.6975
Difficulty thinking	50.00	43.75	0.5642
Impaired sleep or daytime sleepiness	75.41	66.67	0.3933
Back, chest, or abdominal weakness	48.33	56.25	0.4436
Problems with shoulders or arms	50.82	37.50	0.1802
Numbness	70.49	66.67	0.6827
Choking or swallowing issues	29.51	22.92	0.5155
Abnormal movements	55.74	47.92	0.4453
Problems with hands or fingers	50.82	35.42	0.1233
Breathing difficulties	16.67	10.42	0.4113
Seizures	5.00	8.33	0.6975
Impaired vision	31.67	31.25	1.0000
Difficulty hearing	40.98	18.75	0.0212*

	Sex		
	Prevalence (%)		
	Male (n = 47)	Female (n = 62)	*p* value
*C: Theme*			
Limitations with mobility or walking	91.49	83.87	0.2660
Problems with balance	93.62	88.71	0.5100
Inability to do activities	93.62	74.19	0.0100*
Trouble getting around	91.49	62.9	0.0006*
Leg weakness	93.62	80.65	0.0896
Pain	72.34	75.81	0.8252
Stiffness	89.36	80.65	0.2889
Fatigue	87.23	85.48	1.0000
Gastrointestinal issues	63.83	64.52	1.0000
Decreased satisfaction in social situations	80.85	67.74	0.1887
Emotional issues	80.85	75.81	0.6425
Communication difficulties	46.81	37.10	0.3319
Difficulty thinking	48.94	45.90	0.8464
Impaired sleep or daytime sleepiness	65.96	75.81	0.2891
Back, chest, or abdominal weakness	59.57	45.90	0.1782
Problems with shoulders or arms	40.43	48.39	0.4419
Numbness	74.47	64.52	0.3019
Choking or swallowing issues	14.89	35.48	0.0174*
Abnormal movements	61.70	45.16	0.1212
Problems with hands or fingers	36.17	50.00	0.1755
Breathing difficulties	10.64	16.39	0.5759
Seizures	12.77	1.64	0.0414*
Impaired vision	25.53	36.07	0.2981
Difficulty hearing	25.53	35.48	0.3019

	Education level		
	Prevalence (%)		
	High school, Technical school, none (n = 52)	College, Masters, Doctorate (n = 57)	*p* value
*D: Theme*			
Limitations with mobility or walking	88.46	85.96	0.7794
Problems with balance	94.23	87.72	0.3257
Inability to do activities	88.46	77.19	0.1375
Trouble getting around	80.77	70.18	0.2674
Leg weakness	88.46	84.21	0.5864
Pain	82.69	66.67	0.0787
Stiffness	86.54	82.46	0.6063
Fatigue	94.23	78.95	0.0261*
Gastrointestinal issues	61.54	66.67	0.6896
Decreased satisfaction in social situations	78.85	68.42	0.2792
Emotional issues	76.92	78.95	0.821
Communication difficulties	40.38	42.11	1
Difficulty thinking	47.06	47.37	1
Impaired sleep or daytime sleepiness	78.85	64.91	0.1378
Back, chest, or abdominal weakness	56.86	47.37	0.342
Problems with shoulders or arms	55.77	35.09	0.0354*
Numbness	63.46	73.68	0.3026
Choking or swallowing issues	30.77	22.81	0.3904
Abnormal movements	59.62	45.61	0.18
Problems with hands or fingers	51.92	36.84	0.1263
Breathing difficulties	15.69	12.28	0.7815
Seizures	5.88	7.02	1
Impaired vision	37.25	26.32	0.2996
Difficulty hearing	36.54	26.32	0.3026

	Employment status		
	Prevalence (%)		
	Not working (n = 24)	Working (n = 85)	*p* value
*E: Theme*			
Limitations with mobility or walking	95.83	84.71	0.2968
Problems with balance	100.00	88.24	0.1134
Inability to do activities	87.50	81.18	0.5586
Trouble getting around	87.50	71.76	0.1794
Leg weakness	87.50	85.88	1.0000
Pain	95.83	68.24	0.0068*
Stiffness	95.83	81.18	0.1124
Fatigue	100.00	82.35	0.0383*
Gastrointestinal issues	66.67	63.53	0.8149
Decreased satisfaction in social situations	79.17	71.76	0.6040
Emotional issues	100.00	71.76	0.0016*
Communication difficulties	58.33	36.47	0.0638
Difficulty thinking	60.87	43.53	0.1628
Impaired sleep or daytime sleepiness	83.33	68.24	0.2020
Back, chest, or abdominal weakness	70.83	46.43	0.0397*
Problems with shoulders or arms	58.33	41.18	0.1660
Numbness	87.50	63.53	0.0265*
Choking or swallowing issues	29.17	25.88	0.7957
Abnormal movements	75.00	45.88	0.0195*
Problems with hands or fingers	50.00	42.35	0.6421
Breathing difficulties	25.00	10.71	0.0952
Seizures	8.33	5.95	0.6501
Impaired vision	33.33	30.95	0.8084
Difficulty hearing	16.67	35.29	0.1328

	Disability status		
	Prevalence (%)		
	On disability (n = 16)	Not on disability (n = 93)	*p* value
*F: Theme*			
Limitations with mobility or walking	100.00	84.95	0.2163
Problems with balance	100.00	89.25	0.3524
Inability to do activities	87.50	81.72	0.7331
Trouble getting around	87.50	73.12	0.3481
Leg weakness	87.50	86.02	1.0000
Pain	93.75	70.97	0.0655
Stiffness	100.00	81.72	0.0711
Fatigue	100.00	83.87	0.1205
Gastrointestinal issues	62.50	64.52	1.0000
Decreased satisfaction in social situations	75.00	73.12	1.0000
Emotional issues	100.00	74.19	0.0204*
Communication difficulties	68.75	36.56	0.0258*
Difficulty thinking	53.33	46.24	0.7815
Impaired sleep or daytime sleepiness	81.25	69.89	0.5494
Back, chest, or abdominal weakness	62.50	50.00	0.4232
Problems with shoulders or arms	56.25	43.01	0.4170
Numbness	81.25	66.67	0.3816
Choking or swallowing issues	43.75	23.66	0.1248
Abnormal movements	68.75	49.46	0.1832
Problems with hands or fingers	50.00	43.01	0.7860
Breathing difficulties	25.00	11.96	0.2318
Seizures	12.50	5.43	0.2766
Impaired vision	31.25	31.52	1.0000
Difficulty hearing	18.75	33.33	0.3816

	Has ALD impacted your employment status or choice?		
	Prevalence (%)		
	Yes (n = 60)	No (n = 40)	*p* value
*G: Theme*			
Limitations with mobility or walking	93.33	77.50	0.0321*
Problems with balance	95.00	85.00	0.1505
Inability to do activities	93.33	67.50	0.0011*
Trouble getting around	88.33	60.00	0.0015*
Leg weakness	91.67	80.00	0.1287
Pain	88.33	55.00	0.0003*
Stiffness	91.67	72.50	0.0134*
Fatigue	90.00	80.00	0.2387
Gastrointestinal issues	66.67	60.00	0.5291
Decreased satisfaction in social situations	85.00	55.00	0.0013*
Emotional issues	90.00	55.00	< 0.0001*
Communication difficulties	55.00	20.00	0.0008*
Difficulty thinking	61.02	32.50	0.0076*
Impaired sleep or daytime sleepiness	80.00	55.00	0.0135*
Back, chest, or abdominal weakness	64.41	32.50	0.0022*
Problems with shoulders or arms	55.00	25.00	0.0039*
Numbness	80.00	52.50	0.0045*
Choking or swallowing issues	31.67	17.50	0.1625
Abnormal movements	68.33	27.50	< 0.0001*
Problems with hands or fingers	51.67	32.50	0.0672
Breathing difficulties	16.95	2.50	0.0461*
Seizures	10.17	2.50	0.2361
Impaired vision	35.59	22.50	0.1874
Difficulty hearing	30.00	35.00	0.6642

	Years since first noticed symptoms		
	Prevalence (%)		
	Above mean (> 15 years) (n = 51)	Equal to or below mean (≤ 15 years) (n = 57)	*p* value
*H: Theme*			
Limitations with mobility or walking	92.16	84.21	0.2468
Problems with balance	94.12	89.47	0.4953
Inability to do activities	86.27	78.95	0.4485
Trouble getting around	86.27	66.67	0.0237*
Leg weakness	88.24	84.21	0.5893
Pain	82.35	66.67	0.0797
Stiffness	90.20	78.95	0.1220
Fatigue	90.20	82.46	0.2782
Gastrointestinal issues	70.59	57.89	0.2287
Decreased satisfaction in social situations	76.47	70.18	0.5185
Emotional issues	78.43	77.19	1.0000
Communication difficulties	50.98	33.33	0.0794
Difficulty thinking	54.00	42.11	0.2483
Impaired sleep or daytime sleepiness	76.47	68.42	0.3948
Back, chest, or abdominal weakness	62.00	42.11	0.0528
Problems with shoulders or arms	58.82	31.58	0.0065*
Numbness	74.51	64.91	0.3030
Choking or swallowing issues	37.25	17.54	0.0293*
Abnormal movements	64.71	42.11	0.0217*
Problems with hands or fingers	56.86	31.58	0.0114*
Breathing difficulties	26.00	3.51	0.0014*
Seizures	8.00	5.26	0.7031
Impaired vision	30.00	31.58	1.0000
Difficulty hearing	39.22	24.56	0.1459

	Diagnosed with AMN?		
	Prevalence (%)		
	Yes (n = 71)	No (n = 27)	*p* value
*I: Theme*			
Limitations with mobility or walking	94.37	70.37	0.0030*
Problems with balance	95.77	85.19	0.0886
Inability to do activities	88.73	66.67	0.0161*
Trouble getting around	81.69	62.96	0.1974
Leg weakness	91.55	77.78	0.0852
Pain	81.69	59.26	0.0339*
Stiffness	92.96	70.37	0.0063*
Fatigue	87.32	85.19	0.7487
Gastrointestinal issues	70.42	51.85	0.1000
Decreased satisfaction in social situations	76.06	62.96	0.2135
Emotional issues	78.87	77.78	1.0000
Communication difficulties	45.07	29.63	0.1779
Difficulty thinking	50.00	37.04	0.2673
Impaired sleep or daytime sleepiness	71.83	70.37	1.0000
Back, chest, or abdominal weakness	52.11	51.85	1.0000
Problems with shoulders or arms	46.68	37.04	0.4962
Numbness	76.06	44.44	0.0041*
Choking or swallowing issues	25.35	25.93	1.0000
Abnormal movements	57.75	44.44	0.2638
Problems with hands or fingers	45.07	33.33	0.3621
Breathing difficulties	14.08	11.11	1.0000
Seizures	5.63	3.70	1.0000
Impaired vision	21.13	44.44	0.0409
Difficulty hearing	25.35	40.74	0.1462

	Diagnosed with cerebral ALD?		
	Prevalence (%)		
	Yes (n = 18)	No (n = 81)	*p* value
*J: Theme*			
Limitations with your mobility or walking	83.33	86.42	0.7152
Problems with balance	77.78	92.59	0.0800
Inability to do activities	88.89	80.25	0.5138
Trouble getting around	77.78	74.07	1.0000
Leg weakness	77.78	86.42	0.4653
Pain	66.67	76.54	0.3826
Stiffness	77.78	86.42	0.4653
Fatigue	83.33	86.42	0.7152
Gastrointestinal issues	66.67	62.96	1.0000
Decreased satisfaction in social situations	83.33	72.94	0.5495
Emotional issues	88.89	77.78	0.5158
Communication difficulties	50	37.04	0.4243
Difficulty thinking	50	46.25	0.7994
Impaired sleep or daytime sleepiness	66.67	71.6	0.7758
Back, chest, or abdominal weakness	55.56	48.75	0.7948
Problems with shoulders or arms	50	41.98	0.6038
Numbness	72.22	67.9	0.7868
Choking or swallowing issues	22.22	27.16	0.7743
Abnormal movements	66.67	50.62	0.2975
Problems with hands or fingers	44.44	44.44	1
Breathing difficulties	16.67	11.25	0.6899
Seizures	11.11	6.25	0.609
Impaired vision	38.89	26.25	0.3861
Difficulty hearing	22.22	30.86	0.5748

	Diagnosed with Addison's disease (adrenal insufficiency)?		
	Prevalence (%)		
	Yes (n = 35)	No (n = 71)	*p* value
*K: Theme*			
Limitations with mobility or walking	88.57	85.92	1.0000
Problems with balance	94.29	88.73	0.4913
Inability to do activities	91.43	77.46	0.1068
Trouble getting around	91.43	66.20	0.0046*
Leg weakness	91.43	83.10	0.3756
Pain	68.57	76.06	0.4841
Stiffness	85.71	83.10	1.0000
Fatigue	88.57	84.51	0.7688
Gastrointestinal issues	62.86	64.79	1.0000
Decreased satisfaction in social situations	77.14	70.42	0.4984
Emotional issues	80.00	76.06	0.8061
Communication difficulties	42.86	38.03	0.6762
Difficulty thinking	48.57	44.29	0.6842
Impaired sleep or daytime sleepiness	65.71	73.24	0.4975
Back, chest, or abdominal weakness	57.14	49.30	0.5365
Problems with shoulders or arms	28.57	50.70	0.0379*
Numbness	77.14	64.79	0.2655
Choking or swallowing issues	17.14	30.99	0.1626
Abnormal movements	65.71	45.07	0.0627
Problems with hands or fingers	40.00	46.48	0.5419
Breathing difficulties	14.29	14.08	1.0000
Seizures	14.29	2.82	0.0381*
Impaired vision	31.43	32.39	1.0000
Difficulty hearing	28.57	32.39	0.8243

	Ambulatory status		
	Prevalence (%)		
	Mobility assistance needed (n = 59)	Walk independently (n = 50)	*p* value
*L: Theme*			
Limitations with mobility or walking	98.31	74.00	0.0002*
Problems with balance	100.00	80.00	0.0002*
Inability to do activities	100.00	62.00	< 0.0001*
Trouble getting around	98.31	48.00	< 0.0001*
Leg weakness	96.61	74.00	0.0007*
Pain	81.36	66.00	0.0808
Stiffness	93.22	74.00	0.0077*
Fatigue	91.53	80.00	0.0991
Gastrointestinal issues	64.41	64.00	1.0000
Decreased satisfaction in social situations	83.05	62.00	0.0169*
Emotional issues	81.36	74.00	0.3661
Communication difficulties	50.85	30.00	0.0329*
Difficulty thinking	51.72	42.00	0.3394
Impaired sleep or daytime sleepiness	74.58	68.00	0.5247
Back, chest, or abdominal weakness	63.79	38.00	0.0117*
Problems with shoulders or arms	49.15	40.00	0.4398
Numbness	74.58	62.00	0.2132
Choking or swallowing issues	32.20	20.00	0.1932
Abnormal movements	62.71	40.00	0.0217*
Problems with hands or fingers	42.37	46.00	0.8466
Breathing difficulties	13.79	14.00	1.0000
Seizures	10.34	2.00	0.1200
Impaired vision	34.48	28.00	0.5360
Difficulty hearing	33.90	28.00	0.5401

	Speech status		
	Prevalence (%)		
	Speech change (n = 20)	Talks clearly (n = 89)	*p* value
*M: Theme*			
Limitations with mobility or walking	100.00	84.27	0.0685
Problems with balance	100.00	88.76	0.2028
Inability to do activities	95.00	79.78	0.1883
Trouble getting around	100.00	69.66	0.0030*
Leg weakness	100.00	83.15	0.0682
Pain	85.00	71.91	0.2713
Stiffness	95.00	82.02	0.1897
Fatigue	90.00	85.39	0.7335
Gastrointestinal issues	75.00	61.80	0.3119
Decreased satisfaction in social situations	95.00	68.54	0.0222*
Emotional issues	100.00	73.03	0.0059*
Communication difficulties	100.00	28.09	< 0.0001*
Difficulty thinking	73.68	41.57	0.0126*
Impaired sleep or daytime sleepiness	95.00	66.29	0.0117*
Back, chest, or abdominal weakness	78.95	46.07	0.0113*
Problems with shoulders or arms	75.00	38.2	0.0052*
Numbness	95.00	62.92	0.0061*
Choking or swallowing issues	75.00	15.73	< 0.0001*
Abnormal movements	80.00	46.07	0.0067*
Problems with hands or fingers	75.00	37.08	0.0026*
Breathing difficulties	42.11	7.87	0.0007*
Seizures	5.26	6.74	1.0000
Impaired vision	47.37	28.09	0.1110
Difficulty hearing	35.00	30.34	0.7903

	Functional ability		
	Prevalence (%)		
	Slight through severe disability (n = 79)	No symptoms or no significant disability (n = 30)	*p* value
*N: Theme*			
Limitations with mobility or walking	98.73	56.67	< 0.0001*
Problems with balance	97.47	73.33	0.0005*
Inability to do activities	98.73	40.00	< 0.0001*
Trouble getting around	94.94	23.33	< 0.0001*
Leg weakness	96.2	60.00	< 0.0001*
Pain	84.81	46.67	0.0001*
Stiffness	92.41	63.33	0.0005*
Fatigue	93.67	66.67	0.0008*
Gastrointestinal issues	68.35	53.33	0.1807
Decreased satisfaction in social situations	84.81	43.33	< 0.0001*
Emotional issues	83.54	63.33	0.0367*
Communication difficulties	51.90	13.33	0.0002*
Difficulty thinking	55.13	26.67	0.0099*
Impaired sleep or daytime sleepiness	77.22	56.67	0.0555
Back, chest, or abdominal weakness	61.54	26.67	0.0013*
Problems with shoulders or arms	54.43	20.00	0.0013*
Numbness	77.22	46.67	0.0048*
Choking or swallowing issues	29.11	20.00	0.4674
Abnormal movements	64.56	20.00	< 0.0001*
Problems with hands or fingers	48.10	33.33	0.1983
Breathing difficulties	15.38	10.00	0.5518
Seizures	8.97	0.00	0.1866
Impaired vision	34.62	23.33	0.3556
Difficulty hearing	30.38	33.33	0.8187

	Home health aide per week		
	Prevalence (%)		
	Receive aide (n = 34)	None (n = 75)	*p* value
*O: Theme*			
Limitations with mobility or walking	100.00	81.33	0.0046*
Problems with balance	100.00	86.67	0.0292*
Inability to do activities	100.00	74.67	0.0006*
Trouble getting around	100.00	64.00	< 0.0001*
Leg weakness	94.12	82.67	0.1395
Pain	82.35	70.67	0.2414
Stiffness	97.06	78.67	0.0199*
Fatigue	97.06	81.33	0.0340*
Gastrointestinal issues	70.59	61.33	0.3947
Decreased satisfaction in social situations	88.24	66.67	0.0199*
Emotional issues	91.18	72.00	0.0265*
Communication difficulties	52.94	36.00	0.1410
Difficulty thinking	52.94	44.59	0.5340
Impaired sleep or daytime sleepiness	79.41	68.00	0.2585
Back, chest, or abdominal weakness	72.73	42.67	0.0062*
Problems with shoulders or arms	61.76	37.33	0.0226*
Numbness	76.47	65.33	0.2733
Choking or swallowing issues	38.24	21.33	0.1001
Abnormal movements	76.47	41.33	0.0008*
Problems with hands or fingers	55.88	38.67	0.1014
Breathing difficulties	18.18	12.00	0.3837
Seizures	18.18	1.33	0.0031*
Impaired vision	33.33	30.67	0.8241
Difficulty hearing	38.24	28.00	0.3723

*Values of *p* < 0.05 are marked by an asterisk, and values of statistical significance, by the Benjamini–Hochberg method, are bolded

Individuals who reported having anywhere from a slight to severe disability, compared to no symptoms or no significant disability, showed higher prevalence of 14 symptomatic themes; the most significant differences (*p* < 0.0001) were in limitations with mobility or walking (99% vs. 57%), inability to do activities (99% vs. 40%), trouble getting around (95% vs. 23%), leg weakness (96% vs. 60%), decreased satisfaction in social situations (85% vs. 43%), and abnormal movements (65% vs. 20%). Functional ability was the single most closely associated clinical feature with symptomatic theme prevalence.

Similarly, considering speech status, those who indicated experiencing a change in their speech had greater frequency in 9 symptomatic themes; the largest differences (*p* < 0.0001) were related to communication difficulties (100% vs. 28% reported) and choking or swallowing issues (75% vs. 16% reported). In terms of ambulatory status, individuals requiring mobility assistance reported higher prevalence in 6 of the 24 symptomatic themes; the leading differences (*p* < 0.0001) were related to inability to do activities (100% vs. 62% reported) and trouble getting around (98% vs. 48% reported). Receiving home health aide was also highly associated with greater symptomatic theme prevalence in 6 areas, with the most significant difference (*p* < 0.0001) in trouble getting around (100% vs. 64% reported).

Unemployed participants displayed a higher frequency of pain and emotional issues compared to employed participants. When participants were asked if ALD/AMN impacted their employment status or choice, the subgroup of individuals who responded “yes” showed higher prevalence in 11 of the 24 symptomatic themes; the most significant differences (*p* < 0.0001) were in emotional issues (90% vs. 55% reported) and abnormal movements (68% vs. 28% reported).

Participants who had been experiencing symptoms related to ALD for above the mean duration of 15 years reported a higher frequency in problems with shoulders or arms and breathing difficulties. Individuals who were diagnosed with AMN reported a higher prevalence of limitations with mobility or walking, stiffness, and numbness. Individuals who were diagnosed with Addison’s disease experienced a higher frequency of trouble getting around.

Participants older than the mean age of 51 years experienced a higher prevalence of limitations with mobility or walking, compared to participants at or below the mean age. Men experienced trouble getting around at a higher rate than women. There were no significant associations between symptomatic theme prevalence and education level, disability status, or diagnosis with cerebral ALD.

## Discussion

This research provides a novel data set and analysis regarding symptomatic disease in ALD, thereby adding to existing knowledge of ALD and its core clinical manifestations. This information can be used by researchers, therapeutic developers, clinicians, and patients who seek to better understand ALD (and all of the phenotypes) from the patient’s point of view. In this study, qualitative interviews were conducted, in which individuals with ALD identified numerous problematic symptoms that affect their lives. The subsequent cross-sectional study, with a large, international cohort of adults with ALD determined the prevalence and impact of these symptoms and themes.

In ALD, the symptomatic themes with the highest prevalence were also those with the highest relative impact: problems with balance, limitations with mobility or walking, and leg weakness. The overlap of most prevalent issues with issues that are most impactful is seen in some, but not all, diseases [20, 25–27]. Moreover, the importance of these themes in ALD corroborates existing literature [29, 30]. Raymond et al. [29] report the symptom set that affects 40–45% of individuals with ALD (specifically, AMN) to include progressive stiffness and weakness in the legs, and Percy and Rutledge [30] report that boys with ALD (specifically, cerebral ALD) typically present with neurological deterioration that includes development of quadriparesis. In our cross-sectional study, we observed that 54.1% of respondents used some kind of ambulation assistance (cane, crutches, walker, wheelchair, or motorized scooter), and that 84.4% and 86.2% of respondents had stiffness and leg weakness, respectively.

Furthermore, Winkelman et al. [31] show, through diagnositic phone interviews with 32 patients and chart reviews, that progressive gait and balance problems, leg discomfort, pain, and sleep disturbances (related to restless leg syndrome) are highly prevalent and interconnected in adults with ALD. Indeed, we found 90.8% of participants had problems with balance, 74.3% had pain, 71.6% had impaired sleep or daytime sleepiness, and 74.1% had restless legs. Corre et al. [32] present that, in addition to gait and balance issues, bladder and bowel issues are very common in adults with ALD; in their cross-sectional study of 109 adults with ALD, 76.9% of participants had experienced at least one bladder symptom, and 67.3% had experienced at least one bowel symptom. In our study, 86.1% of participants reported trouble with bladder control, and 66.3% reported trouble with bowel control.

Subgroup analysis provides insight into how specific symptomatic themes differ in prevalence based on the characteristics of individuals with ALD; these are general associations and do not indicate a causal relationship. Female participants with ALD (specifically, AMN) communicated trouble getting around at a lower frequency than male participants. The fact that only up to 80% of females develop any kind of symptoms related to ALD during their lifetimes and most of those who do get symptoms experience them after the age of 40–60 years, with the clinical course being less severe, likely explains the lower prevalence of symptoms when compared to men [33, 34]. Participants above the mean age of 51 years showed greater frequency in problems related to mobility and walking. This worsening of physical symptoms related to the spinal cord and peripheral nerves, particularly motor disability of the lower limbs, spasticity, and pain, as individuals get older is consistent with the clinical classification and prognosis of ALD (specifically, AMN) as a progressive disorder [9, 29, 30, 35].

In the analysis of those who identified as having AMN (as opposed to those without AMN), three symptomatic themes were found to be more common: limitations with mobility or walking, stiffness, and numbness. Indeed, these are hallmark symptom areas of AMN, as confirmed by the literature, and these areas need to be appropriately addressed when caring for patients with this condition. [7, 12] In the examination of those who said they have Addison’s disease (as opposed to those without Addison’s disease), one symptomatic theme was found to be more recurrent: trouble getting around. This may relate to the fatigue and muscle weakness that are recognized as cardinal signs of this condition [17]. In the investigation of those with the cerebral form of ALD, we did not find any significant differences in symptomatic theme prevalence, despite the literature denoting cognitive and behavioral impairements, vision problems, and seizures as more common in this cohort [13]. The difference in findings may be attributed to the small sample of patients with cerebral ALD in our study, such that statistical changes could not be detected.

Interestingly and as shown in several other studies with similar methodology to ours, employment status, especially change in employment, had a significant association with symptomatic theme prevalence [19, 20, 22–27]. In our cross-sectional study cohort, not working was associated with higher frequency of 2 symptomatic themes, and a change in employment status or choice due to ALD was associated with higher symptomatic burden in 11 areas. As effective therapies are developed for ALD, it is possible that these therapies will not only reduce individual patient burden but also allow for more open, productive, and meaningful employment opportunities.

We found that the prevalence of two symptomatic themes was associated with a longer time since the onset of symptoms: problems with shoulders or arms and breathing difficulties. In the clinical setting, these two symptomatic themes should be monitored for progression and may be worthy targets for therapeutic interventions [29, 30, 35].

Some of the most widespread differences in symptomatic theme prevalence were seen in those who reported functional disability, those who had speech changes, those who required mobility assistance, and those who received home health aide. The etiology behind the interconnectedness between these concepts and patient-reported symptomatic burden is worth further exploration during future studies.

We acknowledge that there are limitations to this research. The large cohort of individuals with ALD who participated in the cross-sectional study is not a perfect representation of the larger ALD patient population. Although more than 100 adults with all phenotypes of ALD provided data, these participants were limited to those enrolled in one of the national or international registries used for recruitment. In addition, participants self-reported their diagnoses in this study, and these diagnoses were not verified by the registries or by the researchers. Although AMN, cerebral ALD, Addison’s disease, and asymptomatic women with ALS are all considered subsets of ALD, some participants may have misinterpreted their condition. For example, some participants may have had AMN but considered themselves to have ALD only (without AMN) if that was the term commonly used by their physician; or vice versa, some participants may have had AMN and thought of themselves to have AMN only (and not ALD) if they were unfamiliar with the classification of AMN as a subtype of ALD.

Study participants could have differed from the broader ALD patient population in that they represented those with moderate disease burden; asymptomatic individuals or those with very severe symptoms may not have had the willingness or capability to engage in this study. Relatedly, the inclusion of some asymptomatic women with ALD may have diluted the average symptomatic burden found in women overall [34], and the inclusion of younger individuals (more of whom would be asymptomatic or presymptomatic) may have lessened the overall disease burden reported for the entire sample [9].

Our study cohort also included a high percent of participants who identified as white (89.9%) and non-Hispanic (86.2%), and far fewer from minority races and ethnicities. The lack of minority participation in research is a longstanding challenge but one that our clinical research team and organization are working to address through continued and more diversified outreach in the community.

Our recruitment and cross-sectional survey study were conducted primarily online, so individuals without email or access to the internet were also probably underrepresented. Nevertheless, the results from our study do likely reflect the responses for the section of the ALD population that is likely to seek care and participate in research and clinical trials in the future.

## Conclusions

This research significantly adds to existing literature that explores the unique symptoms and co-morbidities of adult patients of both sexes living with any phenotypic variant of ALD, encompassing AMN, cerebral ALD, Addison’s disease, and asymptomatic women with ALD. This study uses extensive and direct patient input to identify what is most meaningful to patients with ALD overall and differences in the symptoms that are most important to distinct subgroups of patients with ALD. The information presented further highlights the multifactorial nature of ALD, and it has implications for identifying clinically-relevant symptoms to address during clinical care and future therapeutic studies.

### Supplementary Information

**Additional file 1**. Prevalence, average life impact, and population impact (PIP) of symptoms inquired about in ALD cross-sectional study (n = 109).

## Data Availability

Anonymized data used and analyzed during the current study are available from the corresponding author on reasonable request.
