# Supplementary material for: Patient-reported impact of symptoms in adrenoleukodystrophy (PRISM-ALD)
Source: Orphanet J Rare Dis. 2024 Mar 19;19:127. doi: 10.1186/s13023-024-03129-6 (PMC10953228; doi:10.1186/s13023-024-03129-6)
Supplement: Supplementary file 1 — Additional file 1. Prevalence, average life impact, and population impact (PIP) of symptoms inquired about in ALD cross-sectional study (n = 109). [file 13023_2024_3129_MOESM1_ESM.docx]

**Patient-Reported Impact of Symptoms in Adrenoleukodystrophy (PRISM-ALD)**

**Supplemental Content**

**Table S1. Prevalence, average life impact, and population impact (PIP) of symptoms inquired about in ALD cross-sectional study (n=109)**

| **Symptom** | **Prevalence (%)** | **Average Impact (0-4)** | **Population Impact (0-4)** |
| --- | --- | --- | --- |
| *Limitations with mobility or walking* | 87.20 | 2.12 | 1.84 |
| *Problems with balance* | 90.80 | 2.21 | 2.01 |
| *Inability to do activities* | 82.60 | 2.12 | 1.75 |
| *Trouble getting around* | 75.20 | 2.34 | 1.76 |
| *Leg weakness* | 86.20 | 2.24 | 1.94 |
| *Pain* | 74.30 | 1.72 | 1.28 |
| *Stiffness* | 84.40 | 1.91 | 1.61 |
| *Fatigue* | 86.20 | 1.95 | 1.68 |
| *Gastrointestinal issues* | 64.20 | 1.79 | 1.15 |
| *Decreased satisfaction in social situations* | 73.40 | 1.59 | 1.17 |
| *Emotional issues* | 78.00 | 1.51 | 1.17 |
| *Communication difficulties* | 41.30 | 0.96 | 0.39 |
| *Difficulty thinking* | 47.20 | 1.02 | 0.48 |
| *Impaired sleep or daytime sleepiness* | 71.60 | 1.47 | 1.06 |
| *Back, chest, or abdominal weakness* | 51.90 | 1.45 | 0.75 |
| *Problems with shoulders or arms* | 45.00 | 1.22 | 0.55 |
| *Numbness* | 68.80 | 1.45 | 1.00 |
| *Choking or swallowing issues* | 26.60 | 1.07 | 0.28 |
| *Abnormal movements* | 52.30 | 1.44 | 0.75 |
| *Problems with hands or fingers* | 44.00 | 1.21 | 0.53 |
| *Breathing difficulties* | 13.90 | 0.80 | 0.11 |
| *Seizures* | 6.50 | 1.14 | 0.07 |
| *Impaired vision* | 31.50 | 1.09 | 0.34 |
| *Difficulty hearing* | 31.20 | 0.82 | 0.26 |
| Impaired walking | 83.20 | 2.38 | 1.98 |
| Difficulty going up stairs | 83.20 | 2.22 | 1.85 |
| Difficulty going down stairs | 83.20 | 2.21 | 1.84 |
| Difficulty running | 89.60 | 2.94 | 2.63 |
| Difficulty playing sports | 84.90 | 3.06 | 2.59 |
| Difficulty riding a bike | 79.00 | 2.89 | 2.29 |
| Fear of falling | 78.50 | 2.42 | 1.90 |
| Difficulty standing for a long time | 86.00 | 2.33 | 2.00 |
| Tripping | 85.80 | 2.36 | 2.03 |
| Falls | 77.40 | 2.16 | 1.67 |
| Difficulty getting up quickly | 86.00 | 2.15 | 1.85 |
| Difficulty moving quickly | 83.20 | 2.38 | 1.98 |
| Difficulty changing direction while walking | 74.50 | 2.47 | 1.84 |
| Having to hold on to things when walking | 75.70 | 2.62 | 1.98 |
| Difficulty walking up hills or inclines | 80.40 | 2.51 | 2.02 |
| Problems with coordination | 80.20 | 1.80 | 1.44 |
| Trouble standing with eyes closed | 84.00 | 2.21 | 1.86 |
| Needing to plan ahead due to physical limitations | 80.80 | 2.11 | 1.70 |
| Difficulty cleaning a house | 75.00 | 2.09 | 1.57 |
| Difficulty maintaining your personal hygiene | 43.30 | 1.60 | 0.69 |
| Difficulty showering or bathing | 50.00 | 1.71 | 0.86 |
| Difficulty washing your hair | 39.40 | 1.71 | 0.67 |
| Trouble getting in or out of a tub or shower | 66.30 | 1.68 | 1.12 |
| Difficulty dressing yourself | 49.00 | 1.51 | 0.74 |
| Difficulty putting on your shoes | 58.70 | 1.51 | 0.88 |
| Difficulty getting on and off of a toilet | 49.00 | 1.63 | 0.80 |
| Difficulty cutting nails | 48.50 | 1.66 | 0.81 |
| Trouble exercising | 78.60 | 1.99 | 1.56 |
| Difficulty preparing food | 51.00 | 1.74 | 0.88 |
| Difficulty with household chores | 69.20 | 1.64 | 1.13 |
| Difficulty washing dishes | 46.20 | 1.81 | 0.84 |
| Difficulty getting in and out of vehicles | 73.10 | 1.47 | 1.08 |
| Difficulty changing lightbulbs | 59.80 | 2.43 | 1.45 |
| Difficulty dancing | 71.60 | 2.82 | 2.02 |
| Difficulty lifting a glass to drink | 20.40 | 1.14 | 0.23 |
| Difficulty turning pages | 17.60 | 0.83 | 0.15 |
| Difficulty using car/house keys | 20.20 | 1.19 | 0.24 |
| Erectile dysfunction (if N/A, please mark first column) | 28.40 | 2.79 | 0.79 |
| Difficulty getting around the house | 63.40 | 1.83 | 1.16 |
| Difficulty using and moving legs | 79.00 | 2.24 | 1.77 |
| Difficulty getting out of a chair | 79.20 | 1.76 | 1.40 |
| Difficulty getting up from the floor or ground | 90.10 | 2.27 | 2.05 |
| Hip, thigh, or knee weakness | 77.00 | 2.14 | 1.65 |
| Difficulty standing | 78.80 | 1.90 | 1.49 |
| Legs giving out | 79.00 | 1.97 | 1.56 |
| Shaky legs | 78.00 | 1.72 | 1.34 |
| Muscle spasms | 82.00 | 1.85 | 1.52 |
| Muscle pain with activity | 72.00 | 1.69 | 1.22 |
| Limited activity due to pain | 65.30 | 1.91 | 1.25 |
| Muscle cramps | 71.00 | 1.58 | 1.12 |
| Back pain | 71.30 | 1.92 | 1.37 |
| Neck pain | 48.50 | 1.57 | 0.76 |
| Headaches | 40.60 | 1.22 | 0.50 |
| Spasticity of muscles | 83.20 | 2.11 | 1.75 |
| Tight muscles | 82.20 | 2.12 | 1.74 |
| Impaired endurance | 83.20 | 2.12 | 1.76 |
| Fatigue after physical activity | 89.10 | 2.10 | 1.87 |
| Muscle fatigue | 85.10 | 2.03 | 1.73 |
| Tired muscles | 86.10 | 1.91 | 1.64 |
| Muscle exhaustion | 79.20 | 1.96 | 1.55 |
| Trouble with bladder control | 86.10 | 2.08 | 1.79 |
| Trouble with bowel control | 66.30 | 2.04 | 1.36 |
| Trouble getting to the bathroom in time | 77.20 | 2.06 | 1.59 |
| Frequent bathroom visits during the day | 80.20 | 2.11 | 1.69 |
| Having to frequently go to the bathroom at night | 78.00 | 2.04 | 1.59 |
| Increased frequency of urination | 80.00 | 1.99 | 1.59 |
| Trouble initiating urination | 57.00 | 2.11 | 1.20 |
| Bladder or bowel dysfunction | 68.70 | 2.31 | 1.59 |
| Constipation | 62.00 | 1.69 | 1.05 |
| Nausea | 26.70 | 0.78 | 0.21 |
| Needing assistance to go to the bathroom | 17.00 | 2.12 | 0.36 |
| Diarrhea | 35.00 | 1.29 | 0.45 |
| Vomiting | 5.90 | 0.50 | 0.03 |
| Decreased independence | 72.00 | 2.00 | 1.44 |
| Reliance on friends and family | 69.00 | 2.09 | 1.44 |
| Impaired sexual function | 57.70 | 2.59 | 1.49 |
| Lack of public awareness or knowledge of ALD or AMN | 79.00 | 2.22 | 1.75 |
| Inability to participate in fun activities | 79.00 | 2.18 | 1.72 |
| Lack of information about the disease | 69.00 | 1.83 | 1.26 |
| Difficulty keeping up with friends | 66.00 | 1.94 | 1.28 |
| Impaired interactions with friends | 59.00 | 1.76 | 1.04 |
| Difficulty engaging with others | 56.00 | 1.45 | 0.81 |
| Social isolation | 59.00 | 1.76 | 1.04 |
| Being perceived as drunk when not drinking | 40.00 | 1.85 | 0.74 |
| Difficulty holding children | 48.50 | 1.73 | 0.84 |
| Reduced sex drive | 59.20 | 2.14 | 1.27 |
| Abnormal change of skin color | 26.00 | 0.88 | 0.23 |
| Fear of disease progression | 91.80 | 2.41 | 2.21 |
| Depression | 75.50 | 1.81 | 1.37 |
| Loneliness | 63.60 | 1.54 | 0.98 |
| Decreased confidence | 76.80 | 1.87 | 1.43 |
| Anxiety | 78.80 | 1.81 | 1.42 |
| A feeling of helplessness | 71.70 | 1.65 | 1.18 |
| Frustration | 84.80 | 1.77 | 1.51 |
| Decreased motivation | 77.80 | 1.82 | 1.41 |
| Reduced enjoyment with activities | 75.80 | 1.92 | 1.45 |
| Emotional strain | 80.80 | 1.74 | 1.40 |
| Embarrassment | 66.70 | 1.68 | 1.12 |
| Fear | 70.70 | 1.59 | 1.12 |
| Anger | 59.60 | 1.83 | 1.09 |
| Sadness | 77.80 | 1.68 | 1.30 |
| Moodiness | 69.70 | 1.64 | 1.14 |
| Stress | 83.80 | 1.76 | 1.47 |
| A feeling of loss of control | 74.50 | 1.86 | 1.39 |
| Behavioral problems | 40.80 | 1.20 | 0.49 |
| Difficulty speaking clearly | 32.30 | 1.09 | 0.35 |
| Difficulty getting words out when upset | 41.40 | 1.15 | 0.47 |
| Problems understanding what others are saying | 29.30 | 0.90 | 0.26 |
| Slurred speech | 23.20 | 0.61 | 0.14 |
| Having to repeat oneself when speaking | 32.30 | 0.72 | 0.23 |
| Impaired memory | 61.60 | 1.33 | 0.82 |
| Difficulty reading | 27.60 | 0.96 | 0.27 |
| Problems focusing | 51.50 | 1.14 | 0.59 |
| Problems concentrating | 54.50 | 1.15 | 0.63 |
| Impaired attention | 48.50 | 1.08 | 0.53 |
| Difficulty with comprehension | 36.40 | 1.03 | 0.37 |
| Forgetfulness | 60.60 | 1.23 | 0.75 |
| Difficulty managing your finances | 21.20 | 0.81 | 0.17 |
| Daytime sleepiness | 72.70 | 1.25 | 0.91 |
| Insomnia | 57.60 | 1.60 | 0.92 |
| Difficulty staying asleep | 68.70 | 1.54 | 1.06 |
| Difficulty falling asleep at night | 62.60 | 1.39 | 0.87 |
| Waking up for long periods of time during the night | 52.50 | 1.25 | 0.66 |
| Having to sleep a lot | 52.50 | 1.17 | 0.62 |
| Difficulty bending down | 76.50 | 2.09 | 1.60 |
| Difficulty sitting up from a lying position | 69.40 | 1.82 | 1.27 |
| Weak trunk (core) muscles | 74.50 | 1.68 | 1.26 |
| Difficulty maintaining good posture | 79.60 | 1.73 | 1.38 |
| Difficulty rolling over in bed | 50.50 | 1.94 | 0.98 |
| Difficulty holding your head up while sitting | 21.40 | 1.29 | 0.28 |
| Difficulty reaching for objects overhead | 51.00 | 1.54 | 0.79 |
| Arm weakness | 44.90 | 0.93 | 0.42 |
| Difficulty lifting objects | 57.10 | 1.46 | 0.84 |
| Difficulty pushing yourself up with your arms | 49.00 | 1.42 | 0.69 |
| Burning pain at the feet | 57.10 | 2.04 | 1.16 |
| Loss of sensation | 67.30 | 1.92 | 1.30 |
| A pins and needles sensation at the skin | 63.30 | 1.60 | 1.01 |
| A tingling sensation at the skin | 58.20 | 1.74 | 1.01 |
| Trouble swallowing | 26.50 | 0.58 | 0.15 |
| Difficulty swallowing liquids | 16.30 | 0.56 | 0.09 |
| Difficulty swallowing food | 22.40 | 0.64 | 0.14 |
| Choking | 16.30 | 0.75 | 0.12 |
| Uncontrolled muscle movements | 66.00 | 1.52 | 1.00 |
| Restless legs | 74.20 | 1.75 | 1.30 |
| Twitching | 58.80 | 1.67 | 0.98 |
| Tremors | 42.30 | 1.46 | 0.62 |
| Voice tremors | 17.50 | 0.94 | 0.16 |
| Difficulty doing things with your hands | 36.10 | 1.09 | 0.39 |
| Finger weakness | 37.10 | 0.81 | 0.30 |
| Dropping objects with your hands | 45.40 | 1.27 | 0.58 |
| Difficulty opening jars or bottles | 54.60 | 1.08 | 0.59 |
| Problems cutting food | 22.70 | 0.86 | 0.20 |
| Difficulty using eating utensils | 15.50 | 0.80 | 0.12 |
| Difficulty using buttons or zippers | 30.90 | 1.07 | 0.33 |
| Difficulty writing with a pen or pencil | 30.90 | 1.10 | 0.34 |
| Shortness of breath | 25.80 | 1.00 | 0.26 |
| Shortness of breath with activities of daily living | 28.90 | 1.00 | 0.29 |
| Shortness of breath while speaking | 19.60 | 1.16 | 0.23 |
